# Persistent DNA Damage Foci and DNA Replication with a Broken Chromosome in the African Trypanosome

**DOI:** 10.1128/mBio.01252-19

**Published:** 2019-07-09

**Authors:** Lucy Glover, Catarina A. Marques, Olga Suska, David Horn

**Affiliations:** a Wellcome Trust Centre for Anti-Infectives Research, School of Life Sciences, University of Dundee, Dundee, United Kingdom; Harvard T. H. Chan School of Public Health

**Keywords:** RPA2, damage response, telomere

## Abstract

Damaged DNA typically imposes stringent controls on eukaryotic cell cycle progression, ensuring faithful transmission of genetic material. Some DNA breaks, and the resulting rearrangements, are advantageous, however. For example, antigenic variation in the parasitic African trypanosome, Trypanosoma brucei, relies upon homologous recombination-based rearrangements of telomeric variant surface glycoprotein (*VSG*) genes, triggered by breaks. Surprisingly, trypanosomes with a severed telomere continued to grow while progressively losing subtelomeric DNA, suggesting a nominal telomeric DNA damage checkpoint response. Here, we monitor the single-stranded DNA-binding protein replication protein A (RPA) in response to induced, locus-specific DNA breaks in T. brucei. RPA foci accumulated at nucleolar sites following a break within ribosomal DNA and at extranucleolar sites following a break elsewhere, including adjacent to transcribed or silent telomeric *VSG* gene*s.* As in other eukaryotes, RPA foci were formed in S phase and γH2A and RAD51 damage foci were disassembled prior to mitosis. Unlike in other eukaryotes, however, and regardless of the damaged locus, RPA foci persisted through the cell cycle, and these cells continued to replicate their DNA. We conclude that a DNA break, regardless of the damaged locus, fails to trigger a stringent cell cycle checkpoint in T. brucei. This DNA damage tolerance may facilitate the generation of virulence-enhancing genetic diversity, within subtelomeric domains in particular. Stringent checkpoints may be similarly lacking in some other eukaryotic cells.

## Abstract (precis)

Chromosome damage must be repaired to prevent the proliferation of defective cells. Alternatively, cells with damage must be eliminated. This is true of human and several other cell types but may not be the case for single-celled parasites, such as trypanosomes. African trypanosomes, which cause lethal diseases in both humans and livestock, can actually exploit chromosomal damage to activate new surface coat proteins and to evade host immune responses, for example. We monitored responses to single chromosomal breaks in trypanosomes using a DNA-binding protein that, in response to DNA damage, forms nuclear foci visible using a microscope. Surprisingly, and unlike what is seen in mammalian cells, these foci persist while cells continue to divide. We also demonstrate chromosome replication even when one chromosome is broken. These results reveal a remarkable degree of damage tolerance in trypanosomes, which may suit the lifestyle of a single-celled parasite, potentially facilitating adaptation and enhancing virulence.

## INTRODUCTION

Trypanosoma brucei is a protozoan parasite and the causative agent of human African trypanosomiasis, also known as sleeping sickness, and Nagana in livestock, with the human disease being typically fatal without therapy (1). T. brucei is transmitted by the tsetse fly and exists as an extracellular parasite in the mammalian host bloodstream (2) but also resides in adipose tissue (3) and in skin (4, 5). T. brucei cells are covered by a dense layer of a highly immunogenic variant surface glycoprotein (VSG), and it is against this VSG that an immune response is directed. Strict monoallelic expression of a single *VSG* gene from a subtelomeric site and the ability to switch the expressed *VSG* underpin antigenic variation and immune evasion (6). The single active *VSG* gene is transcribed by RNA polymerase I (pol-I) in a monoallelic fashion from a specialized telomeric locus called an expression site (ES) (7). Despite being pol-I driven, *VSG* genes are not transcribed in the nucleolus but rather at a distinct extranucleolar compartment termed the expression site body (ESB) (8, 9). Switching the active *VSG* gene with another *VSG* gene or assembling a new mosaic *VSG* gene from an archive of approximately 2,000 silent *VSG* genes and gene fragments requires RAD51-dependent homologous recombination (HR) or repair by microhomology-mediated end joining (MMEJ) (7), typically initiated by a DNA double-strand break (DSB). This process allows the trypanosomes to produce immunologically distinct surface coats and to continue to escape the host’s immune response. Recombination among silent *VSG* genes may also allow diversification of the *VSG* reservoir available for activation. Indeed, active (10) and silent (11) *VSG* ESs display spontaneous DNA breaks. Notably, T. brucei cells can continue to divide with a damaged silent *VSG* ES (12), and the wider “VSGnome” is remarkably plastic during T. brucei propagation (13); while the diploid chromosomal cores are homozygous, the *VSG*-rich subtelomeric regions are heterozygous (14). The emergence of drug resistance also involves DNA rearrangements (15).

In higher eukaryotes, nonhomologous end joining (NHEJ) is the primary repair pathway operating in the G_1_ phase of the cell cycle, with HR operating in the late S and G_2_ phases (16). NHEJ, however, appears to be absent in trypanosomatids, where HR and MMEJ are the dominant forms of DSB repair (17, 18). Central to HR and MMEJ is the formation of single-stranded DNA (ssDNA) through 5′ end resection, which generates 3′ ssDNA overhangs on either side of a DSB (19). In mammalian cells, this then leads to the recruitment of factors associated with the DNA damage response (DDR), which can go on to trigger a stringent G_2_/M cell cycle checkpoint (20); damage can then be removed before further cell cycle progression. Activation of this DDR may be particularly persistent following damage at telomeres and often results in senescence (21). Experimental monitoring of the cellular DDR is facilitated by the recruitment of repair factors to the site of a DSB (22), and in T. brucei, γH2A and RAD51 have been used as cytological markers; γH2A is a phosphorylated form of histone H2A that accumulates at sites of DSBs (23), while the RAD51 recombinase forms a helical filament on ssDNA, facilitating strand invasion and HR (24). As seen in other eukaryotes (25), DDR-associated γH2A (23), RAD51 (17), and translesion polymerase foci (26) are typically restricted to the S and G_2_ phases in T. brucei, consistent with replication-associated repair.

DNA resection and the production of ssDNA are an early response to DNA damage. In mammalian cells, distinct single-stranded DNA (ssDNA)-binding proteins (ssDNA-BPs) respond early and control DNA-recombination and repair at different loci and at different cell cycle stages, by protecting and marking resected ssDNA. The major ssDNA-binding protein (ssDNA-BP) in yeast and mammalian cells is replication protein A (RPA; also known as replication factor A [RFA]), which both coats and stabilizes resected DNA and regulates resection (27). RPA can then be replaced by RAD51, which facilitates the formation of a presynaptic complex and homologous recombination (25). RPA forms a heterotrimeric complex consisting of RPA1, RPA2, and RPA3 subunits, all of which contain oligonucleotide/oligosaccharide-binding (OB) folds involved in ssDNA binding (28). RPA focus formation is typically restricted to the S and G_2_ phases of the cell cycle in mammals (29). In these cells, an additional ssDNA-BP, hSSB1, is required for genome stability (30), and both yeast and mammalian cells rely on the Ctc1-Stn1-Ten1 ssDNA-BP to facilitate repair at telomeres (31). The only ssDNA-BP orthologues identified in trypanosomatids are the RPA complex components (32). Transcripts encoding the largest RPA1 subunit are cell cycle regulated in Crithidia fasciculata (33), while RPA1 from both Trypanosoma cruzi (34) and *Leishmania* (35) displays affinity for the G-rich telomeric strand *in vitro*; *Crithidia*, T. cruzi, and *Leishmania*, like T. brucei, are all parasitic trypanosomatids.

Studies on DNA repair in trypanosomes provide a distinct perspective on the evolution of the eukaryotic DNA damage response, beyond the vertebrate, fungal, and other model organisms. They also provide insights into genetic recombination strategies in important human parasites. Following induction of precise chromosomal breaks in T. brucei, we observe RPA DNA damage foci, which form in S phase and, regardless of the site of the break, persist through the cell cycle, without blocking DNA replication. We suggest that this unusual damage tolerance and capacity for replication with a broken chromosome in parasitic trypanosomes facilitate the generation of virulence-enhancing genetic diversity, within subtelomeric domains in particular.

## RESULTS

### RPA foci are detected at all T. brucei cell cycle stages.

**FIG 1:**
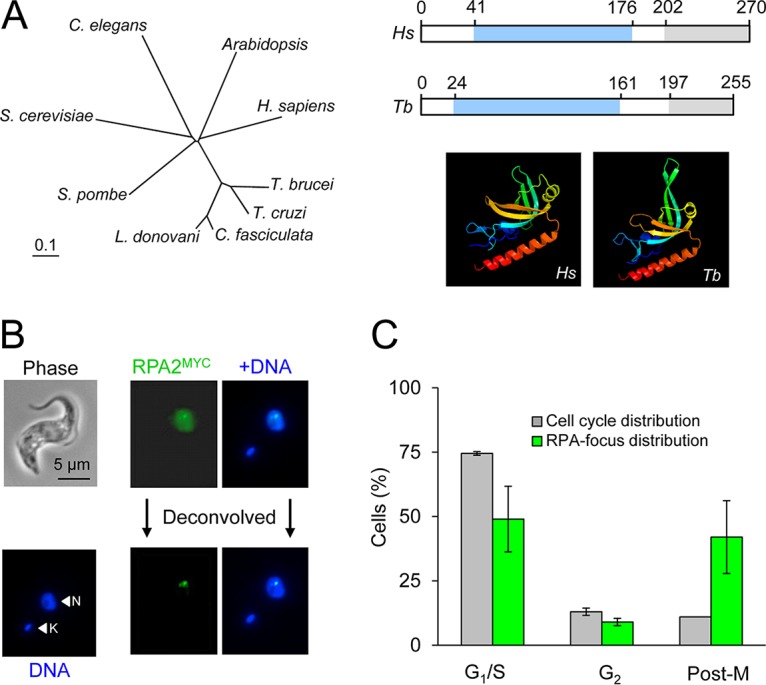
RPA foci are detected at all major cell cycle stages in T. brucei. (A) Phylogenetic analysis. The unrooted neighbor-joining tree was generated using Clustal 1.8X and TreeView. Homo sapiens (NP_001284487.1); Trypanosoma brucei (Tb927.5.1700); Trypanosoma cruzi (TcCLB.510821.50); *Crithidia fasciculata* (CFAC1_060019500); Leishmania donovani (LdBPK_150310.1); Arabidopsis thaliana (NP_566188.1); Saccharomyces cerevisiae (EGA77243.1); Caenorhabditis elegans (CCD64225.1). The schematic shows human (*Hs*) RPA2 and the T. brucei (*Tb*) orthologue. The location of the conserved DNA-binding OB fold (blue) and winged helix-turn-helix (gray) are indicated. The structure of the RPA2 OB fold domain was predicted using Phyre2 and is compared to the human structure. (B) Immunofluorescence microscopy reveals spontaneous formation of RPA foci (0.8% of cells). RPA2^MYC^, green; DNA counterstained with DAPI, blue; N, nucleus; K, kinetoplast. (C) Cell cycle distribution of spontaneous RPA foci. *n* = 200 for cell cycle analysis; *n* = 75 for RPA focus analysis. Error bars, SD for biological replicates; *n* = 2.

*VSG* recombination and gene conversion are critical for antigenic variation in T. brucei, but our understanding of the DNA repair processes involved and the factors influencing repair template selection remains incomplete. We sought to characterize the DSB response in more detail and also sought a cytological marker for improved detection of sites of DSBs. T. brucei RPA1 (Tb927.11.9130), RPA2 (Tb927.5.1700), and RPA3 (Tb927.9.11940) orthologues have been identified, and we selected RPA2 for epitope tagging since this subunit is often used as a cytological marker for DNA damage foci in mammalian cells (36). RPA2 is conserved among trypanosomatids (Fig. 1A, left panel) and, although diverged relative to other eukaryotes, contains a predicted DNA-binding OB fold and a winged helix-turn-helix domain (Fig. 1A, right side, upper panel). Indeed, the OB fold domains are predicted to form similar structures in both the T. brucei and human proteins (37) (Fig. 1A, right side, lower panel). In addition, phosphoproteome data indicate that T. brucei RPA2, like mammalian RPA2 (38), can be phosphorylated at Ser^4^ (39).

We generated a strain expressing a native *RPA2* gene fused to a C-terminal epitope tag. Immunofluorescence analysis revealed diffuse nuclear RPA2^MYC^ staining in all cells and a focal accumulation of RPA2 (Fig. 1B) in approximately 0.8% of cells (*n* = 2,180; 17 cells with foci), presumably reflecting spontaneous DNA breaks. Notably, those cells with RPA foci were distributed across all cell cycle phases examined, and postmitotic cells with foci were 4-fold overrepresented (Fig. 1C). This does not match the pattern of γH2A and RAD51 foci, which predominate in the S and G_2_ phases of the T. brucei cell cycle, presumably as a consequence of DNA damage associated with DNA replication (17, 23). Nor does it match the pattern of RPA2 foci in mammalian cells, which also predominate in the S and G_2_ phases (29).

### RPA foci form in response to induced DSBs at pol-I and pol-II transcribed loci.

**FIG 2:**
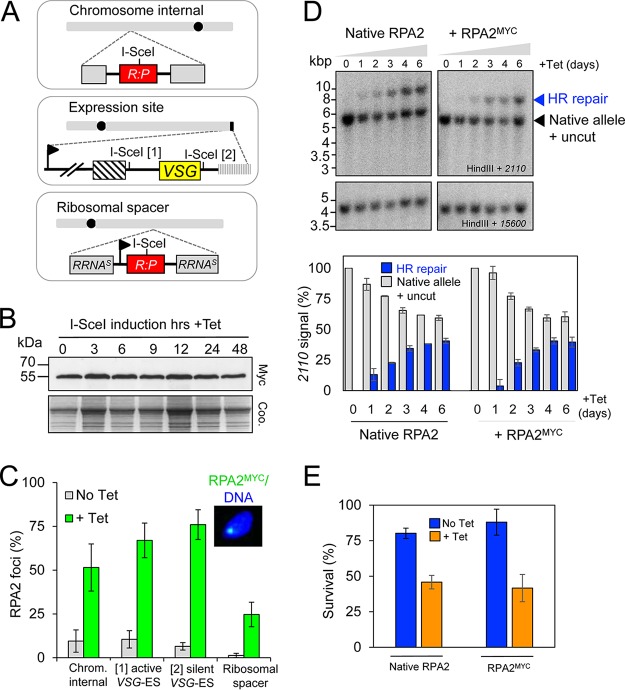
RPA foci form in response to DNA DSBs induced at different sites. (A) Schematic maps indicating the locations of the DSBs induced in this study. (B) RPA2^MYC^ levels remain constant during DSBR. Western blotting with anti-MYC and protein samples extracted at different times after I-SceI induction. An equivalent Coomassie blue-stained gel serves as a loading control. The predicted mass of *Tb*RPA2^MYC^ is 45.1 kDa. (C) RPA foci accumulate at subnuclear foci in response to an induced DSB. Proportions of nuclei with foci were counted in uninduced cells (No Tet) and 12 h after I-SceI induction (+ Tet). *n* = 200 at each time point. Error bars, SD for technical replicates, *n* = 2; with biological replicates, *n* = 2. (D) Monitoring repair by Southern blot analysis. Genomic DNA extracted at various times following I-SceI induction was digested with HindIII and subjected to Southern blot analysis using the probes indicated. Arrowheads indicate comigration of native allele and uncut allele and accumulation of the HR-repaired allele. Probe 2110 was a 699-bp SacI fragment from pARD (71); probe 15600 was a 731-bp HindIII/XhoI coding region fragment of Tb927.11.15600 and served as a loading control. The plot shows quantitative analysis of the blots shown plus an additional strain. Error bars, SD for technical replicates, *n* = 2; and with biological replicates for the RPA2^MYC^ strains, *n* = 2. (E) A clonogenic assay reveals the proportions of cells that survive a DSB at the chromosome-internal locus in the presence of either native RPA2 or RPA2^MYC^. Error bars are as in panel D.

Since RPA2 staining presented an opportunity to monitor DNA damage throughout the cell cycle, we next expressed RPA2^MYC^ in T. brucei cells with an inducible I-SceI meganuclease gene and with a meganuclease cleavage site. We placed a cleavage site at a pol-II transcribed chromosome-internal locus (17), at the active pol-I transcribed *VSG* ES between the *VSG* and the recombinogenic upstream 70-bp repeats (“[1]”) (11), at a silent *VSG* ES downstream of the *VSG* (“[2]”) (12), and at a ribosomal DNA (rDNA) locus (40) (Fig. 2A). Inclusion of the latter locus allowed us to explore the response to DNA damage in a second pol-I-associated subnuclear compartment, the nucleolus. Meganuclease induction did not have a detectable impact on the quantity of RPA2 expressed (Fig. 2B; chromosome-internal locus) but did have a major impact on the proportion of cells with nuclear RPA foci (Fig. 2C). Proportions of cells with RPA foci increased dramatically after 12 h of induction in every case: to approximately 50 to 80% at either the pol-II transcribed locus or at the active or silent *VSG* ES and to approximately 25% after induction of a break at an rDNA locus. The increased number of foci detected prior to induction in some strains, relative to the wild-type background, likely reflects low-level, “leaky” expression of the meganuclease, while reduced accessibility at the rDNA locus likely results in a relatively low proportion of nuclei with RPA foci in these strains. We conclude that focal accumulation of RPA2 in T. brucei involves the redistribution of protein already present in the cell. Thus, all DSBs tested induce focal accumulation of RPA2; those at a pol-II transcribed locus in the chromosome core, at pol-I transcribed or silent subtelomeric *VSG* ESs, and at a pol-I transcribed locus in the nucleolus. Notably, there appears to be no barrier to forming RPA foci within the compact chromatin compartments formed by silent subtelomeric *VSG* genes (14), and these cells continue to divide without repairing the break (12). Thus, RPA foci do not themselves serve as a trigger for a stringent DNA damage checkpoint in T. brucei.

We next used quantitative Southern blotting to determine the efficiency of the break and repair cycle at the chromosome internal locus and to determine whether this was similar in cells expressing RPA2^MYC^. A HindIII polymorphism at this locus allows us to measure HR-dependent repair over time (17). This revealed a break-and-repair cycle that was close to completion in the population after approximately 4 days in the presence of either native RPA2 or RPA2^MYC^ (Fig. 2D). A clonogenic assay also revealed similar survival rates of approximately 50% following a chromosome-internal DSB in the presence of either native RPA2 or RPA2^MYC^ (Fig. 2E). We conclude that neither induced breaks nor DNA repair by HR is perturbed in the presence of RPA2^MYC^.

### RPA foci are detected at all cell cycle stages in response to induced DSBs.

**FIG 3:**
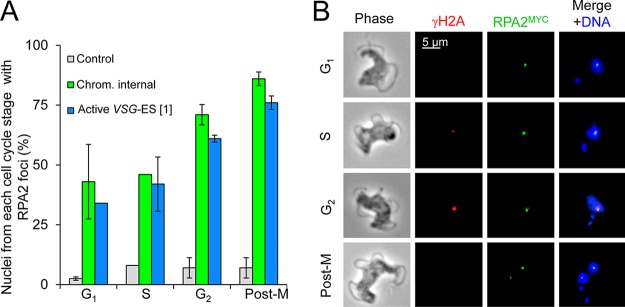
RPA foci are detected at all cell cycle phases. (A) Cell cycle distribution of cells with RPA foci formed 12 h after I-SceI induction. Control, uninduced cells. *n* = 200 for each data point. Error bars, SD for biological replicates; *n* = 2. (B) Immunofluorescence microscopy analysis of γH2A (red) and RPA foci (green). The gallery of representative images shows foci in cells with breaks at the active *VSG* ES. We obtained similar results for cells with breaks at a chromosome-internal locus.

We next examined the cell cycle distribution of RPA foci following induction of a DSB at a chromosome-internal locus or at the active *VSG* ES. As suspected, we detected induced RPA foci at all stages of the cell cycle examined (Fig. 3A). Detection of foci in approximately 40% of G_1_- and S-phase cells, compared to approximately 70% of G_2_ and postmitotic cells, corresponds to the replication of the locus with the I-SceI target site and the presence of two potential breaks in the latter phases. Indeed, postmitosis, both daughter nuclei often contained RPA foci (Fig. 3B). which may reflect RPA accumulation at new DSBs or the persistence and segregation of damaged DNA during mitosis. As expected, we found that the RPA foci were associated with γH2A damage foci in S phase and G_2_ (Fig. 3B); γH2A foci are not typically detected at G_1_ or in postmitotic cells (23).

### Nucleolus-associated RPA foci following a break within ribosomal DNA.

**FIG 4:**
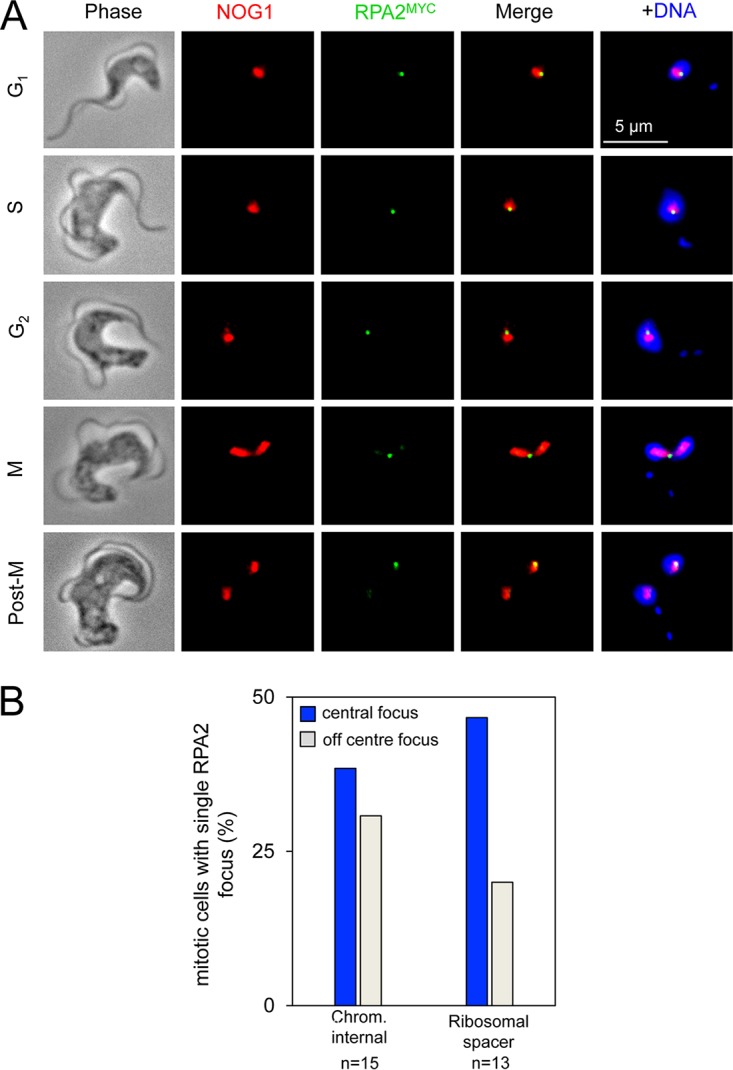
RPA foci are nucleolar following breaks induced at an rDNA locus. Immunofluorescence microscopy analysis of RPA foci 12 h after inducing a break. (A) The gallery of representative images shows cells with RPA foci 12 h after I-SceI induction. NOG1 (nucleolar marker), red; RPA foci, green. Cell cycle phases are indicated. (B) Quantitative analysis of RPA focus distribution in mitotic cells.

We next took advantage of the ability to detect DNA damage foci at all cell cycle stages to monitor the response to damage in the nucleolus. Although T. brucei ribosomal DNA (rDNA) loci are distributed across several chromosomes, they form a single prominent nucleolar compartment. As above, induction of a DSB at an rDNA locus produced RPA foci at all stages of the cell cycle examined (Fig. 4A). These foci coincided with nucleolar staining, except in mitotic cells, where they often sat between dividing nuclei (Fig. 4B and row M in Fig. 4A); RPA foci between dividing nuclei were also observed in cells with a DSB at the pol-II transcribed chromosome-internal locus (Fig. 4B), implying late partitioning of damaged DNA in both cases. In addition, damage-associated nucleolar blebs were observed in G_2_ cells (Fig. 4A, row G_2_). Notably, mammalian RPA damage foci are observed at the nucleolar periphery in G_1_ following induced breaks within rDNA (41) while extranucleolar RPA damage foci were reported following induced breaks within rDNA in Saccharomyces cerevisiae (42).

### Extranucleolar RPA foci following breaks at pol-I transcribed or silent *VSG* ESs.

**FIG 5:**
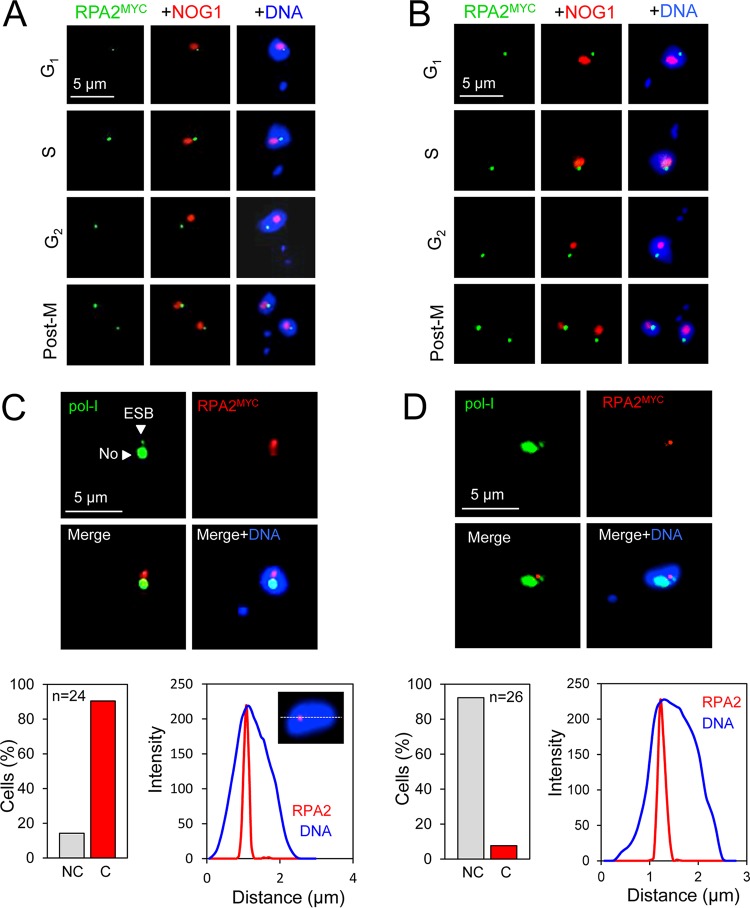
RPA foci following breaks induced at active or silent *VSG* ESs. Immunofluorescence microscopy analysis of RPA foci 12 h after inducing a break. (A) The gallery of representative images shows cells with a break at the active *VSG* ES. RPA foci, green; nucleolar marker (NOG1), red. Cell cycle phases are indicated. (B) As in panel A, but for a silent *VSG* ES. (C) The images show a G_1_ cell with a break at the active *VSG* ES. RPA foci, red; pol-I, green. The ESB and nucleolus (No) are indicated. The lower panels show proportions of cells with RPA and ESB foci that are noncoincident (NC) or coincident (C) (bar graph) and RPA foci in relation to nuclear DNA (linear intensity plot, *n* = 10 G_1_ nuclei). Intensity measurements were taken as indicated in the inset. (D) As in panel C but for a silent *VSG* ES. For the intensity plot, *n* = 9 G_1_ nuclei.

The active subtelomeric *VSG* ES, like the rDNA locus, is transcribed by pol-I, but at an extranucleolar site known as the ESB (8, 9). Silent *VSG* ESs are also extranucleolar in bloodstream-form cells (43), and antigenic variation typically involves replacement of the transcribed *VSG* gene with a new *VSG* gene from the silent archive. We examined RPA foci in cells with breaks at the active *VSG* ES or silent *VSG* ES and, in both cases, detected RPA foci at extranucleolar sites at all stages of the cell cycle (Fig. 5A and B).

Both the ESB and the nucleolus can be labeled using antibodies to pol-I subunits (8). We therefore examined the relationship between *VSG*-associated damage foci and the ESB. RPA foci associated with a DSB at the active *VSG* ES were primarily coincident with the ESB (Fig. 5C, upper panels and bar graph); we specifically surveyed G_1_ cells in this case to minimize complications associated with the appearance of nascent nucleoli. Thus, pol-I is retained at the ESB, rather than dispersed, following a DSB and the formation of RPA DNA damage foci at the active *VSG* ES. These RPA foci associated with breaks at active *VSG* ESs occupied a subnuclear space that was primarily extranucleolar but not associated with the nuclear periphery (Fig. 5C, upper panels and intensity plot). A similar analysis in cells with a DSB at a silent *VSG* ES revealed RPA foci that were noncoincident with the ESB (Fig. 5D, upper panels and bar graph). Although not associated with the ESB, these RPA foci surveyed in G_1_ cells also occupied a subnuclear space that is extranucleolar but not associated with the nuclear periphery (Fig. 5D, upper panels and intensity plot).

### RPA foci associated with a developmentally inactivated *VSG* ES are at the nuclear periphery.

**FIG 6:**
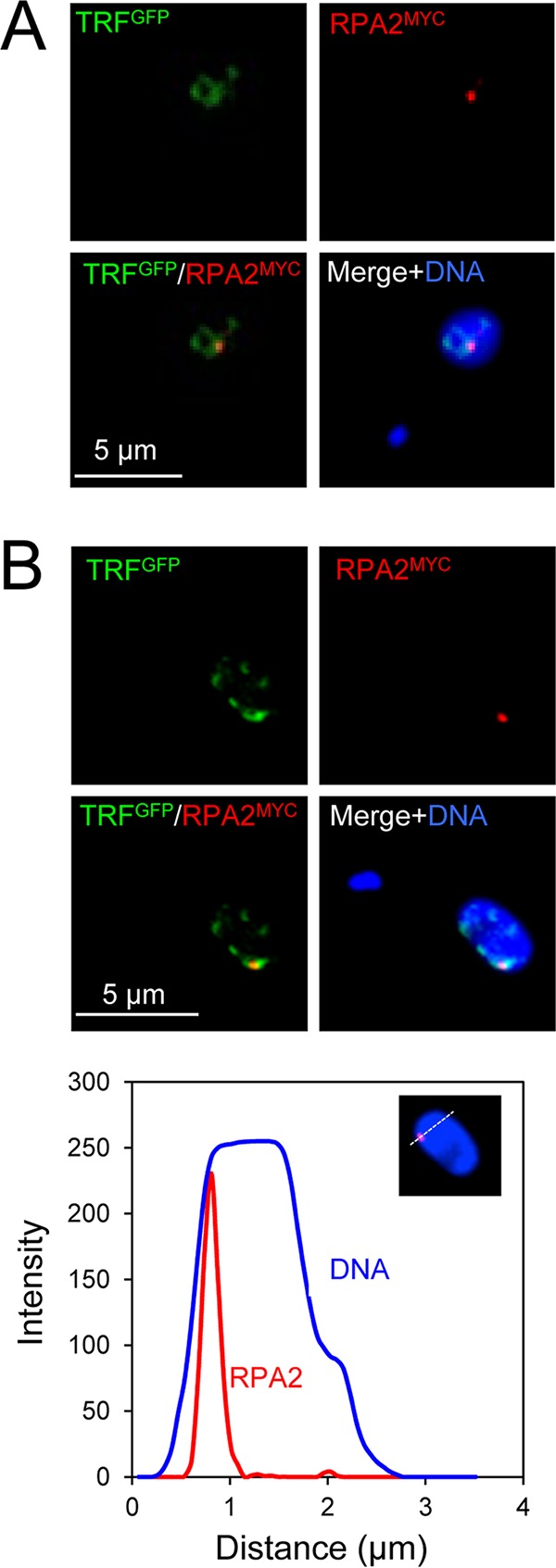
RPA foci at *VSG* ESs in two major life cycle stages of T. brucei. Immunofluorescence microscopy analysis of TRF and RPA foci. (A) Representative images showing the telomeric repeat binding factor (TRF) and focal accumulation of RPA in a G_1_ bloodstream-form T. brucei cell 12 h after DSB induction at the active *VSG* ES. (B) As in panel A, but for a previously active *VSG* ES in insect-stage T. brucei. The lower panel shows a linear intensity plot of RPA foci in relation to nuclear DNA. *n* = 16. Inset, intensity measurements were taken as indicated in the inset.

Upon differentiation following transmission from the mammalian to the insect host, the active *VSG* ES undergoes developmental silencing and repositioning to the nuclear periphery (44). Indeed, the available evidence indicates that *VSG* ESs and subtelomeric DNA relocalize to the nuclear periphery during or soon after differentiation, based on tagging the active (43) or silent (44) *VSG* ESs, by detection of telomeric T_2_AG_3_ repeats (45, 46) or ES-flanking 50-bp repeats (9, 46) or by monitoring localization of the telomere-binding protein TRF (47). To explore RPA foci at this developmentally regulated locus following chromatin condensation, bloodstream-form cells with an I-SceI cleavage site at the active *VSG* ES were differentiated *in vitro*, followed by analysis of meganuclease-induced RPA foci in relation to TRF. As expected, foci associated with the active *VSG* ESs in bloodstream-form cells were found within TRF-associated telomeric clusters (Fig. 6A). RPA foci associated with the inactivated *VSG* ES in insect-stage cells were also colocalized with telomeric clusters but, in this case, at the nuclear periphery (Fig. 6B). Thus, RPA foci still form in response to a DSB at a locus that has undergone developmental silencing and chromatin condensation at the nuclear periphery.

### RPA foci form in S phase and subsequently persist.

**FIG 7:**
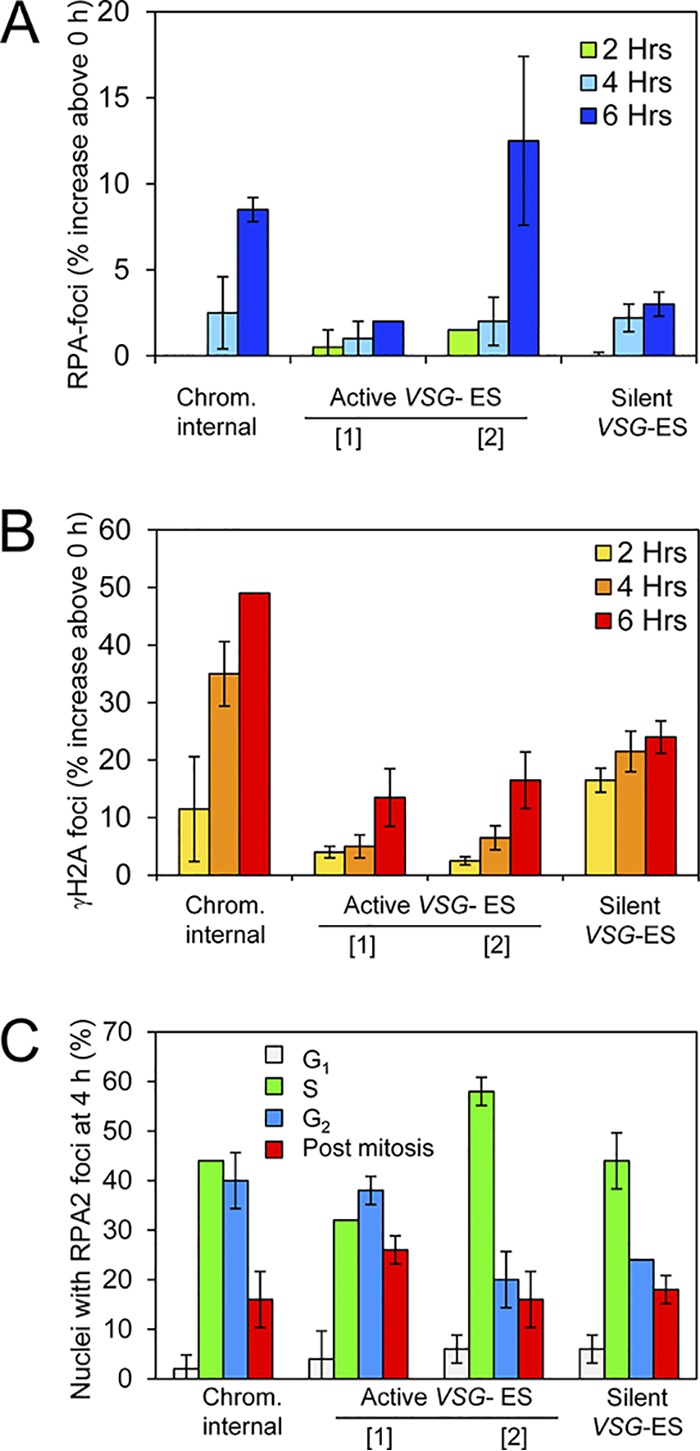
Cell cycle distribution of RPA foci formed immediately after induction of DNA DSBs. (A) Increase in proportions of nuclei with RPA foci at the time points indicated and after DSB induction at the sites indicated. Two distinct break sites were tested at the active *VSG* ES, between the *VSG* and the recombinogenic upstream 70-bp repeats (marked as “[1]”) or downstream of the *VSG* (marked as “[2]”). *n*  = 200 nuclei at each time point. One cell division cycle takes approximately 6 h. (B) As in panel A but for γH2A foci. (C) Cell cycle distribution of RPA foci formed 4 h after induction of DSBs at the sites indicated. *n* = 25 RPA foci in each case. All error bars, SD for biological replicates; *n* = 2.

Detection of RPA foci at all cell cycle stages tested in T. brucei does not reflect the situation observed in other eukaryotes. To determine whether RPA focus assembly is cell cycle stage specific, we monitored the appearance of RPA foci at early time points following the induction of the I-SceI nuclease; γH2A foci were also monitored in parallel. In this case, we tested T. brucei cells with inducible DNA breaks at the pol-II transcribed chromosome-internal locus, at the active pol-I transcribed *VSG* ES, either between the *VSG* and the recombinogenic upstream 70-bp repeats or downstream of the *VSG* (11), and at a silent *VSG* ES downstream of the *VSG*. The proportion of nuclei with RPA foci (Fig. 7A) and γH2A foci (Fig. 7B) continued to increase during the first 6 h after induction, and we selected the 4-h time point for further analysis, since we saw an increase in the proportion of nuclei with RPA foci above background in all four strains tested at this early time point; one cell cycle takes approximately 6 h. Analysis of cells with RPA foci 4 h after inducing the nuclease revealed a striking bias in the cell cycle distribution (Fig. 7C). Thirty to 60% of cells with RPA foci were in S phase, 20 to 40% were in G_2_, and 15 to 30% were postmitotic, while only approximately 5% or less were in G_1_. Detection of relatively few RPA foci in G_1_ nuclei 4 h after nuclease induction (Fig. 7C) and a greatly increased proportion of RPA foci in G_1_ nuclei 12 h after induction (Fig. 3A) indicated assembly of RPA foci in late G_1_ or in S phase and subsequent persistence.

### DNA replication in T. brucei cells with a broken chromosome.

**FIG 8:**
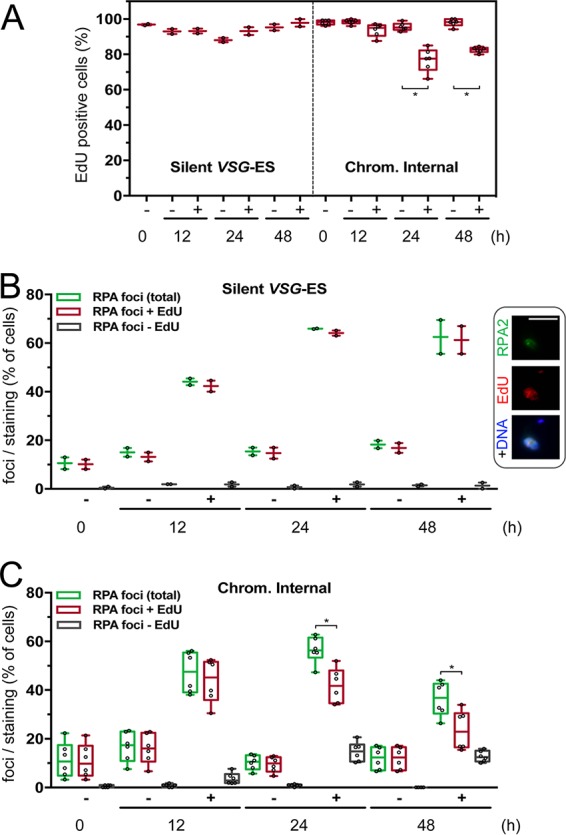
DNA replication in cells with a broken chromosome. (A) The plot shows the percentage of EdU-labeled cells in each population. For silent *VSG* ES, *n* = 2 technical replicates; for Chrom. Internal, *n* = 2 biological and 2 technical replicates and >100 cells for each data point. (B) The plot shows the percentage of cells with a nuclear RPA focus and with or without EdU labeling for the silent *VSG* ES. *n* = 2 technical replicates and >100 cells for each data point. The inset shows an example of a cell with an RPA focus and EdU labeling. Bar, 5 μm. (C) As in panel B but for the chromosome-internal break; *n* = 2 biological and 2 technical replicates and >100 cells for each data point. All plots indicate the interquartile range and median, while the error bars represent the minimum and maximum points. Statistical analysis: repeated-measures one-way analysis of variance (ANOVA), multiple comparisons (matched repeated measurements, compare preselected pairs of columns, Sidak’s multiple-comparison test). *, *P < *0.01.

The results above suggested that T. brucei cells can progress through the cell cycle with a broken chromosome, irrespective of the location of the break. To further investigate this hypothesis, we assessed nuclear DNA replication, in parallel with RPA foci, after DSB induction at a silent *VSG* ES or at a chromosome-internal locus. T. brucei cells expressing RPA2^MYC^ were labeled with the thymidine analogue 5-ethynyl-2′-deoxyuridine (EdU), and both EdU and RPA foci were subsequently detected by fluorescence microscopy (Fig. 8). Prior to induction of DNA breaks, proportions of replicating cells in each population were between 86 and 99%, as assessed by EdU labeling (Fig. 8A). Following DSB induction at the silent *VSG* ES, we saw no reduction in cells undergoing DNA replication at 12, 24, or 48 h (Fig. 8A, left side), and the vast majority of these cells with RPA foci were also labeled with EdU (Fig. 8B). Notably, the proportion of these cells with RPA foci remained high after 48 h, suggesting persistence of ssDNA at the damaged chromosome end. These results are entirely consistent with unperturbed growth with an unrepaired DSB at a silent *VSG* ES (12).

Following DSB induction at the chromosome-internal locus, high proportions of cells remained EdU positive at 12, 24, and 48 h: 87 to 97%, 66 to 85%, and 80 to 85%, respectively. The reduction in cells exhibiting DNA replication was significant at 24 and 48 h but not at 12 h (Fig. 8A, right side). Thus, despite the presence of a DSB, and up to 4 days taken to complete repair (Fig. 2D), DNA replication continued in the majority of these cells. Cells in which DNA replication is perturbed likely represent those cells that ultimately fail to repair the break and die as a result, approximately 50% of the population (Fig. 2E). A high proportion of these cells with RPA foci were also labeled with EdU: 80 to 96%, 62 to 82%, and 51 to 77% at 12, 24, and 48 h, respectively (Fig. 8C). Again, those cells that fail to repair this chromosome-internal break will inevitably contribute to the reduction in EdU labeling at 24 and 48 h, while the reduced frequency of nuclei with RPA foci after 48 h likely reflects successful repair at this locus (Fig. 2D and E). Importantly, the substantial majority of cells with RPA foci also display EdU labeling at all time points tested. These results are consistent with the view that neither subtelomeric nor chromosome-internal DSBs block DNA replication or trigger a stringent cell cycle arrest in T. brucei.

## DISCUSSION

Stringent cell cycle checkpoints in eukaryotic cells minimize the propagation of damaged or rearranged DNA, but some cells, such as protozoan parasites, may not require such stringent controls. Indeed, multiple key cell cycle regulators, conserved among model eukaryotes, are either missing or remain to be identified, present but highly divergent, or functionally replaced by unrelated and phylum-specific factors in trypanosomatids (48–52). This might reflect not only the complex parasitic life cycles of these organisms but also their unique genetic mechanisms.

We have exploited the ssDNA-binding protein RPA as a cytological marker for damaged DNA and find that RPA foci can be used to monitor DSBs at several distinct loci and, surprisingly, at all stages of the cell cycle. Here, the loci tested are transcribed by either pol-I or pol-II, are at chromosome-internal or subtelomeric sites, and are euchromatic or heterochromatic, making RPA foci excellent markers for monitoring DNA breaks at distinct genomic locations in T. brucei. Persistent RPA foci through the cell cycle and continued DNA replication lead us to conclude that an unrepaired DNA break fails to trigger a stringent cell cycle checkpoint in T. brucei, and we suggest that this facilitates the generation of virulence-enhancing genetic diversity.

In T. brucei, homologous recombination and microhomology-mediated end joining dominate DSB repair (17), and both pathways likely contribute to antigenic variation in these parasites (11). We previously reported unabated cell division, despite progressive loss of DNA at a damaged chromosome end in T. brucei (12). Our current findings now indicate that damaged DNA assembles foci of RPA and other repair-associated factors in S phase, following a subtelomeric break or following a chromosome internal break. Only the RPA foci, however, persist through the cell cycle. Thus, African trypanosomes display a remarkable degree of damage tolerance. Genome integrity can be readily restored through homologous recombination when a homologous template is available, and this is likely important to retain essential genes in the chromosomal core regions. Subtelomeric repair appears to be rather inefficient, however, meaning that many cell divisions following damage can produce large numbers of cells with a damaged chromosome end. Notably, this would present many opportunities for repair as a result of a single “founder” DSB, and even low-efficiency repair at subtelomeres has the potential to generate tremendous diversity, since many paralogous templates are available for homologous recombination (53).

Our results indicate that a DSB at either the active or silent *VSG* ES readily accumulates RPA. Whether DSBs lead to transcription inhibition is unknown in trypanosomes. In this respect, it is notable that the accumulation of pol-I at the active *VSG* ES, the ESB, remains intact even when an RPA (this work) or γH2A (23) repair focus is assembled at the same site. Both active and silent *VSG* ESs are extranucleolar, but neither shows any appreciable association with the nuclear periphery in bloodstream-form cells (43). Similarly, we find that broken *VSG* ESs are extranucleolar and not associated with the nuclear periphery in bloodstream-form cells. Upon differentiation from the mammalian stage to the insect tsetse fly stage, the active *VSG* ES rapidly relocalizes from the nuclear interior to the nuclear periphery (44). We find that RPA foci associated with breaks at these sites are also located at the nuclear periphery in insect-stage cells. These data suggest *in situ* assembly of repair factors with no requirement for major relocalization of the DSB. Indeed, this also appears to be the case with ribosomal DNA in trypanosomes, where RPA foci associated with a DSB were perinucleolar, coinciding with the localization of unperturbed ribosomal DNA (44); we also observed perinucleolar blebs associated with DNA damage foci in this case, however.

**FIG 9:**
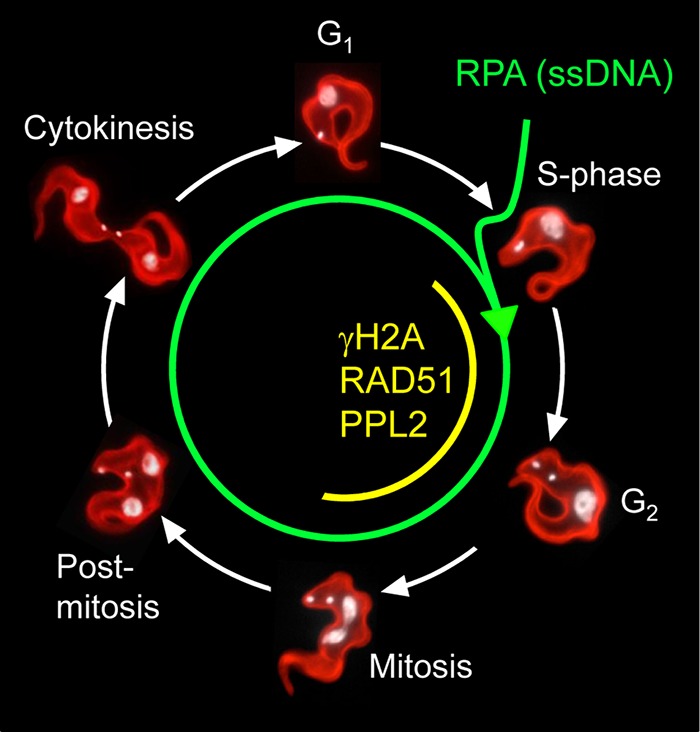
The schematic indicates the origin of ssDNA and RPA foci in S phase and their persistence, in the absence of DNA repair in T. brucei. A DSB *per se* does not trigger a checkpoint. γH2A, RAD51, and PPL2 DNA damage foci display a restricted cell cycle distribution (see the text for more details). Repair likely occurs during the period when these latter factors are assembled, but even in the absence of repair, these latter foci are disassembled prior to mitosis.

Assembly of RPA foci in S phase in T. brucei mirrors observations in other cell types, but persistence of RPA foci through the cell cycle is unusual. The appearance of RPA foci in S phase suggests that resection occurs in S phase. The appearance of γH2A foci (23), RAD51 foci (17), and translesion polymerase foci (26) suggests that repair, as seen in other eukaryotes, typically occurs in the late S and G_2_ phases (Fig. 9). Unlike other eukaryotes, however, neither RPA foci nor failure to repair *per se* is a major impediment to cell cycle progression in trypanosomes. Indeed, a moderate delay observed during late S/G_2_ following a chromosome-internal break (17) might be due to repair by homologous recombination, which is relatively efficient at a chromosome-internal site (17), or the disassembly of γH2A and RAD51 foci. We suspect that relatively few breaks are typically encountered under physiological conditions. Indeed, we detect <1% of normally cultured cells with RPA foci. Perhaps unsurprisingly, multiple DNA breaks trigger a distinct response, as demonstrated in insect-stage T. brucei exposed to ionizing radiation (54). These cells display dramatically increased RAD51 expression, followed by reduced RPA1 expression and an extended period of impaired growth, perhaps also involving persistent damage and loss of essential genes.

Not all previously reported DSBs result in strictly enforced checkpoints. For example, at mammalian telomeres, POT1 inhibits RPA binding, allowing chromosome ends to avoid DNA damage surveillance (55). RPA is also thought to be involved in controlling the G_2_/M checkpoint response in Saccharomyces cerevisiae (56), but these cells can adapt and continue to divide with a DSB after a long (8- to 12-h) Rad9-dependent G_2_/M arrest (57). Divergent components of the 9-1-1 (Rad9-Hus1-Rad1) complex, including Rad9, are present in trypanosomatids, and evidence from *Leishmania* with one Rad9 allele deleted is consistent with a role in delaying cell cycle progression, but only in response to high-frequency chemically induced DSBs (58). In human cells, unreplicated DNA can pass through mitosis (59), and in Drosophila melanogaster, mitotic chromosomal fragments are tethered by DNA bridges, but it is unclear whether this results in the segregation of damaged chromosomes (60). There are also examples of RPA foci that persist through the cell cycle in mammalian cells, specifically those associated with nucleolar DSBs (41) and class switch recombination events (61).

Why might trypanosomes display a high degree of damage tolerance? This may be beneficial for a protozoal pathogen. In the case of African trypanosomes, population bottlenecks occur regularly, and failure to repair breaks would be expected to have minimal negative impact on the population. Indeed, such a strategy may provide a selective advantage, whereby the absence of a checkpoint facilitates *VSG* recombination and switching. There are approximately 20 *VSG* ESs in the trypanosome genome (62), with strict monoallelic expression resulting in a single active *VSG* while the rest are silenced. The subtelomeric *VSG* ESs are fragile and accumulate DSBs independent of transcription status (11). Breaks at a silent *VSG* ES are not associated with a cell cycle checkpoint (12), and we now show that these breaks recruit RPA that persists. This then increases the time available for a homology search (63) and also the number of cells with potential recombination events. Thus, this strategy may increase the diversification of subtelomeric *VSG* loci and contribute to the process of antigenic variation and immune evasion.

When cell cycle progression is uncoupled from DNA damage, repair can still operate, but if it does not, loss of essential genes will lead to cell death. Thus, a predicted genotoxic consequence of a damage tolerance strategy would be loss of essential genes. Consistent with this prediction, approximately 95% of cells fail to recover following an induced break adjacent to the active *VSG* (11) and approximately 50% fail to recover following an induced break at the chromosome-internal locus investigated here. This difference may be partly explained by the action of the T. brucei histone acetyltransferase HAT3, which promotes resection at a chromosome-internal site but restricts resection at subtelomeric sites (64).

RPA appears to be the major ssDNA-binding protein in trypanosomes, and our analysis of this factor in T. brucei has facilitated the monitoring of DNA damage throughout the cell cycle. A stringent cell cycle arrest that is enforced until DNA damage is repaired is important for mammals and other multicellular organisms, since aberrant cells can seed tumors, for example. In contrast, our results in T. brucei indicate a capacity for progression through the cell cycle with a broken chromosome. This may particularly suit a unicellular parasitic lifestyle, since it likely facilitates the generation of genetic diversity, particularly within subtelomeric domains. Thus, while chromosomal breaks are often deleterious, African trypanosomes tolerate them and likely derive an advantage from damage tolerance. This reflects a balance between the dual needs of adaptation and genome stability. Replication with broken chromosomes may be more common than previously appreciated.

## MATERIALS AND METHODS

### Trypanosoma brucei growth and manipulation.

Lister 427, MITat1.2 (clone 221a), bloodstream-form cells were grown in HMI-11 and transformed as described previously (17). Strains expressing TetR and I-SceI with I-SceI cleavage sites at a chromosome-internal locus (17), at an rDNA locus (40), and at active or silent *VSG* ESs (11, 12) were described previously. Cell density was determined using a hemocytometer. Puromycin, phleomycin, G418, hygromycin, and blasticidin were routinely used at 1, 1, 1, 1 and 2 μg.ml^−1,^ respectively. Differentiation to the procyclic stage was triggered *in vitro* by transferring cells to glucose-free DTM supplemented with citrate and *cis*-aconitate at 27°C (65). For the clonogenic assay, ca. 32 cells were distributed across 96-well plates and positive wells were counted 5 to 6 days later.

### Plasmid construction.

For C-terminal epitope tagging at the native pol-II transcribed locus, a Tb927.5.1700/*RPA2* (765-bp) fragment was amplified using primers RPA28F (GATCAAGCTTATGGAAGGAAGTGGAAGTAA) and RPA28R (GATCTCTAGAAATGCCAAACTTACAATCATG) and cloned in pNAT^xTAG^ (66) using the HindIII and XbaI sites (underlined). The construct was linearized with XhoI prior to transfection. For N-terminal epitope tagging of Tb927.10.12850/*TRF*, an 1,146-bp fragment was amplified using primers TFR2FLNtagF (GATCTCTAGATACTGTCACGCTGGCGT) and TFR2FLNtagR (GATCGGATCCTCACTCGTTATTCTCCATATTGG) and cloned in pNAT^TAGx^ (66) using the HindIII and XbaI sites (underlined) and linearized with BlpI prior to transfection. In this case, a *BLA* resistance marker was replaced with an *NPT* resistance marker amplified from p5′NEO5′ and cloned using SpeI and SacI.

### (Immuno)fluorescence microscopy.

Immunofluorescence analysis was carried out using standard protocols as described previously (67). Mouse anti-Myc was used at 1:400, rabbit anti-green fluorescent protein (GFP) was used at 1:500, rabbit anti-pol-I was used at 1:200, rabbit anti-NOG1 (68) was used at 1:500, and rabbit anti-γH2A (23) was used at 1:250. Fluorescein-conjugated goat anti-rabbit and goat anti-mouse secondary antibodies (Pierce) were used at 1:2,000. Samples were mounted in Vectashield (Vector Laboratories) containing 4′,6-diamidino-2-phenylindole (DAPI). In T. brucei, DAPI-stained nuclear and mitochondrial DNA can be used as cytological markers for cell cycle stage (69); one nucleus and one kinetoplast (1N:1K) indicate G_1_, one nucleus and an elongated kinetoplast (1N:eK) indicate S phase, one nucleus and two kinetoplasts (1N:2K) indicate G_2_/M, and two nuclei and two kinetoplasts (2N:2K) indicate postmitosis. Images were captured using an Eclipse E600 microscope with a digital camera (Nikon) and were processed and/or deconvolved in MetaMorph. ImageJ was used to generate linear intensity plots.

### DNA blotting.

Southern blotting was carried out as described previously (17).

### Protein blotting.

Western blotting was carried out according to standard protocols. Mouse anti-Myc was used at 1:2,000. Blots were developed using an ECL kit (Amersham).

### EdU labeling.

Twenty-four hours prior to incubation with 5-ethynyl-2′-deoxyuridine (EdU; Life Technologies, Thermo Scientific), cells were washed and then cultured in thymidine-free medium consisting of Iscove’s modified Dulbecco’s medium (IMDM) (Gibco), 15% (vol/vol) fetal bovine serum (FBS) (Sigma-Aldrich), HMI mix (0.05 mM bathocuproine disulfonic acid, 1 mM sodium pyruvate, and 1.5 mM l-cysteine; Sigma-Aldrich), 1 mM hypoxanthine (Sigma-Aldrich), and 0.0014% 2-mercaptoethanol (Sigma-Aldrich). The cells were maintained in thymidine-free medium for the duration of the experiment. For each time point (0, 12, 24, and 48 h after tetracycline induction), cells were incubated with 150 μM EdU for 4 h at 37°C with 5% CO_2_ (70). Approximately 1 × 10^6^ cells were collected and incubated with 3% formaldehyde (Sigma-Aldrich) in PBS for 15 min (the first 5 min at 37°C and the remaining 10 min at room temperature [RT]), washed twice in 1× PBS, and resuspended in 30 μl 1% bovine serum albumin (BSA) (Sigma-Aldrich). Three microliters of cell suspension was then loaded onto each well of a 12-well multiwell glass slide (Thermo Scientific) and allowed to dry overnight at RT. The cells were then rehydrated in PBS (three times, 5 min each), washed twice in 3% BSA in 1× PBS (5 min each), and permeabilized in 0.2% Triton X-100 (Sigma-Aldrich) in PBS, for 15 min, at RT. The cells were washed with PBS for 5 min, and then with 3% BSA twice (5 min each), before being incubated for 1 h at RT with 25 μl of Click-iT EdU detection mix, protected from light. The detection mix was composed of 21.5 μl of reaction buffer, 1 μl 100 mM CuSO_4_, 0.25 μl Alexa Fluor 555, and 2.5 μl of buffer additive. The cells were then washed five times with 3% BSA (5 min each) and incubated with mouse anti-Myc clone 9B11 antiserum (Cell Signaling Technology), diluted 1:5,000 in 1% BSA in PBS, for 1 h at RT. The slides were then washed three times in PBS and incubated with goat anti-mouse fluorescein isothiocyanate (FITC) conjugate antiserum (Thermo Scientific), diluted 1:100 in 1% BSA in PBS, for 1 h at RT. The cells were washed three times with PBS, dried, and then mounted in Vectashield. Images were acquired in an Axiovert 200M (Zeiss) microscope coupled with an Apotome 2 system and an AxiCam MRm camera, using Zen Blue (Zeiss). Images were then processed using both Zen Blue and Fiji; RPA foci were then quantified from Z-stack images using Fiji. Statistical analyses were performed in GraphPad Prism 7.
